# Transcatheter Mitral Valve Replacement in Failed Bioprosthetic Valve, Ring, and Mitral Annular Calcification Associated Mitral Valve Disease Using Balloon Expandable Transcatheter Heart Valve

**DOI:** 10.14797/mdcvj.1221

**Published:** 2023-05-16

**Authors:** Hiroki A. Ueyama, Patrick T. Gleason, Vasilis C. Babaliaros, Adam B. Greenbaum

**Affiliations:** 1 Emory Structural Heart and Valve Center, Emory University Hospital Midtown, Atlanta, Georgia, US

**Keywords:** transcatheter mitral valve replacement, mitral valve disease, mitral annular calcification

## Abstract

Transcatheter mitral valve replacement (TMVR) using the SAPIEN platform has been performed in failed bioprosthetic valves (valve-in-valve), surgical annuloplasty rings (valve-in-ring), and native valves with mitral annular calcification (MAC) (valve-in-MAC). Experience over the past decade has identified important challenges and solutions to improve clinical outcomes. In this review, we discuss the indication, trend in utilization, unique challenges, procedural planning, and clinical outcomes of valve-in-valve, valve-in-ring, and valve-in-MAC TMVR.

## Introduction

Transcatheter mitral valve replacement (TMVR) using the commercially available SAPIEN platform (Edwards Lifesciences) has evolved as a provisional measure to treat mitral valve disease while dedicated devices are under investigation. TMVR relies on an anatomical landing zone to anchor a valve not specifically designed for the mitral annulus and therefore is mostly performed in patients at high surgical risk with failed bioprosthetic valves (valve-in-valve [ViV]), surgical annuloplasty rings (valve-in-ring [ViR]), or significant mitral annular calcification (MAC) (valve-in-MAC [ViMAC]). The use of TMVR has increased since the first description of ViV TMVR via the transapical approach in 2009, and important lessons have been learned.^1^ This review summarizes the indications, contemporary trends in utilization, unique challenges, pre- and periprocedural planning, and clinical outcomes with contemporary ViV, ViR, and ViMAC TMVR.

## Indication and Trends in Utilization

The use of surgical mitral valve repair has increased over the past decade,^2^ and for patients undergoing mitral valve replacement, the use of bioprosthetic rather than “rigid” valves has increased 3-fold (16.8% in 1996 to 53.7% in 2013).^3^ Consequently, the number of failed bioprosthetic or previously repaired valves requiring re-intervention is expanding. The indication for re-intervention in failing bioprosthetic valves or annuloplasty rings is straightforward: symptomatic patients with severe mitral stenosis, mitral regurgitation, or mixed disease are considered for eligibility. Although outcomes of redo mitral valve surgery have improved, a report from the Society of Thoracic Surgeons (STS) database still documents an operative mortality approaching 11%.^4^ Thus, transcatheter strategies such as ViV and ViR TMVR are attractive alternatives in this scenario. Early reports comparing TMVR to redo surgical mitral valve surgery have demonstrated less major bleeding, new-onset atrial fibrillation, and hospital length of stay after TMVR with no significant difference in periprocedural and 1-year mortality despite significantly higher STS scores.^5,6^

More recently, a report from a large database including 2,745 patients demonstrated substantially lower perioperative mortality in ViV TMVR (ViV TMVR < 2.8% vs redo surgical mitral valve replacement 7.6%).^7^ No randomized controlled trials have compared these two strategies to date. The American and European guidelines recognize ViV TMVR as a reasonable alternative in select patients with increased surgical risk.^8,9^ Reflecting the need from the clinical community and US Food and Drug Administration (FDA)-approval of SAPIEN 3 for use in ViV and ViR TMVR, the report from the STS/American College of Cardiology Transcatheter Valve Therapy (TVT) registry has identified substantial annual volume increases from 84 to 1,120 cases in 2014 and 2019, respectively,^10^ with ViV being the leading indication (78%) followed by ViR (11.7%) and off-label ViMAC (10.3%) TMVR.^10^

Selecting patients with failed bioprosthetic valves for transcatheter intervention is relatively straightforward—they must have appropriately sized valves without major dehiscence. Selection is more complex in those with failed surgical rings due to diversity of ring shapes, sizes, and annular/ventricular anatomy. Valve-in-ring can be performed when there are more than 270 degrees of rigid contact between the transcatheter heart valve and the ring. However, surgical rings with rigid and extreme eccentricity, such as the Edwards GeoForm (Edwards Lifesciences), are not favorable landing zones because they can cause deformation and potential failure of the implanted transcatheter heart valve.^11^ MAC-related mitral valve disease presents even more complex issues and requires careful consideration. Extensive MAC is prevalent in up to 40% of patients with severe mitral stenosis.^12^ These patients are typically elderly, female, and have multiple comorbidities, including frailty and a high prevalence of atherosclerotic risk factors. The transmitral gradient is driven by multiple factors, including the degree of stenosis, left ventricular diastolic dysfunction, reduced left atrial compliance, and mitral regurgitation, which are often present in this population. Therefore, it cannot be used as the sole surrogate of mitral stenosis.^13^ Moreover, symptoms due to comorbid conditions can overlap. Thus, whether correcting the valve problem will provide symptomatic improvement is often difficult to predict, especially in cases of stenosis. The presence of MAC alone, either with or without associated mitral valve disease, is recognized as an important prognostic factor associated with increased mortality.^14^ Surgical intervention for symptomatic MAC is often burdened by associated comorbidities and the technical complexity associated with calcium debridement and reconstruction.^15^ Nonetheless, without intervention, 1-year mortality is reported to be as high as 28%.^12^ Thus, ViMAC TMVR is recognized as a reasonable alternative if symptoms are determined to be primarily related to MAC-associated mitral valve disease after careful evaluation. The use of the SAPIEN 3 device in ViMAC has yet to receive FDA approval, and there is no mention of its recommendation in valvular guidelines. However, its off-label use is steadily increasing, having exceeded that of ViR in 2018.^10^

## Challenges

Compared to a more straightforward transcatheter aortic valve replacement, performing TMVR is challenging because of the complex morphology of the mitral apparatus, which consists of a dynamic 3-dimensional (3D) annulus, anterior and posterior leaflets, chordae tendineae, and two papillary muscles. For TMVR, a bioprosthetic valve provides a rigid landing zone, whereas a surgical ring/band and MAC have much variability in their morphology, rigidity, and distribution, which can lead to unreliable anchoring of TMVR valves. The resultant challenges associated with TMVR include left ventricular outflow tract (LVOT) obstruction, valve embolization and migration, paravalvular leak, valve thrombosis, and unclear longevity.

## Left Ventricular Outflow Tract Obstruction

The LVOT consists of a demarcated area between the basal interventricular septum and intervalvular fibrosa of the aortomitral continuity. Following TMVR, the newly implanted valve frame is covered by the deflected anterior mitral leaflet, creating an elongated narrow “neo-LVOT.”^16^ This often creates LVOT obstruction after TMVR and is a dreaded complication, reaching 54% in-hospital mortality.^17^ The risk of LVOT obstruction after TMVR is the leading cause of screening failure in a contemporary registry consisting of 32% ViMAC and 6% ViR of the total screened cases.^18,19^ The definition of LVOT obstruction varies from registry to registry, ranging from a 10 mm Hg to 30 mm Hg increase in LVOT gradient compared with baseline.^18,19,20,21,22^ Analyzed most conservatively, LVOT obstruction occurs in 7.1% of patients after TMVR, with ViMAC posing a significantly higher rate than ViV and ViR (ViMAC 39.7%, ViV 2.2%, and ViR 5.0%).^20^ Hemodynamically significant LVOT obstruction can be treated by open heart surgery with valve explantation, emergent transcatheter alcohol septal ablation, or bailout anterior leaflet modification (LAMPOON, described below), but the mortality remains high.^17,23,24^ Preprocedural risk assessment and measures to prevent LVOT obstruction are summarized below.

## Valve Embolization and Migration

Valve embolization and migration after TMVR is thought to occur due to the retrograde force from left ventricle systole, inadequate valve sizing, malpositioning of the transcatheter heart valve, and absence of firm anchoring from an annuloplasty ring/band and annular calcification. The STS/TVT registry reported a 0.8% overall incidence of valve embolization (ViV 0.1%, ViR 2.4%, and ViMAC 3%) and 0.4% periprocedural valve migration (ViV 0.3%, ViR 0%, and ViMAC 2%).^25^ Although these incidences vary among registries, they are less frequently observed in ViV TMVR since the implantation is more predictable. The surgical bioprosthesis provides a rigid anchor and the posts often aid valve alignment. Late migration of a transcatheter valve leading to paravalvular and intravalvular regurgitation has also been reported.^26,27^ Treatment of embolization often requires valve snaring and deployment of a second transcatheter heart valve or surgical exploration with transcatheter valve retrieval and redo surgical or hybrid mitral valve replacement.^17,26,27^ Meticulous preprocedural analysis of the landing zone, valve sizing, and intraprocedural evaluation of valve positioning is essential for TMVR success.

### Paravalvular Leak

Paravalvular leak during ViR and ViMAC TMVR typically results from unfavorable surgical ring/band types or valve malpositioning after TMVR. The mechanisms include eccentric and/or rigid (surgical rings) and noncircumferential annuloplasty bands, valve undersizing and underexpansion, asymmetric distribution of MAC, and severe malalignment predicted by excessive angulation between the mitral annulus and left ventricular apex centerline (the “Emory” angle).^11^ The exact incidence of paravalvular leak after TMVR remains unknown due to inconsistent reporting in large registry data. The TMVR registry observed higher periprocedural paravalvular leak closure rates in ViR (ViR 7.8%, ViV 2.2%, and ViMAC 0.0%).^20^ Cases of a paravalvular leak with significant regurgitation can lead to recurrent congestive heart failure and increased mortality, as has been demonstrated in transcatheter aortic valve replacement, and can likely be extrapolated in TMVR.^28^ Increased turbulent flow across the leak is also thought to be related to hemolytic anemia after TMVR and is observed in up to 4% of patients at 30 days.^17,18,19^ Evaluating the etiology, size, and location of paravalvular leaks is challenging and requires multimodality imaging, including 2D and 3D transthoracic or transesophageal echocardiography and cardiac computed tomography (CT). Percutaneous transcatheter paravalvular leak closure can be performed in patients with suitable anatomy.

### Valve Thrombosis and Durability

Longitudinal outcome data for transcatheter aortic valves implanted in the mitral position are scarce. Surgical reports have shown that a bioprosthetic valve in the mitral position is associated with a 2- to 3-times higher incidence of valve thrombosis and shorter longevity compared with those in the aortic position.^29,30^ Subclinical leaflet thrombosis is observed in 12% of patients with aortic valve replacement who had CT at 30 days and appears to occur more commonly in transcatheter versus surgical bioprosthesis.^31,32^ Therefore, theoretically, TMVR may significantly increase the risk of valve thrombosis. Cumulative incidence of valve thrombosis was observed in up to 14.4% of patients after TMVR and appeared more common in patients receiving ViV TMVR and in patients without anticoagulation.^20,21^ The clinical consequences of subclinical valve thrombosis are unclear, and its long-term effect on valve durability, thromboembolic events, and mortality needs further evaluation. The optimal antithrombotic regimen after TMVR has yet to be established, but prospective registries have opted to use lifelong aspirin along with at least 3 months of anticoagulation.^21,22^

## Pre- and Periprocedural Planning

The key to successful TMVR relies on comprehensive multimodality imaging analysis with emphasis on contrast-enhanced, electrocardiography-gated cardiac CT. This allows assessment of the landing zone, determination of valve size, virtual valve implantation, and risk stratification of LVOT obstruction and paravalvular leak.

### Landing Zone and Valve Size

Preprocedural planning is performed at the last phase of systole when the aortic valve is still open. The landing zone is defined by tracing the insertion of the mitral leaflets into the annulus, often creating a 3D ellipsoid in ViMAC.^11^ In ViV and ViR, the most atrial portion of the original prosthesis is traced. Delineation of the landing zone allows careful evaluation of MAC and the surgical ring, including distribution, geometry, dimensions, and compliance. This will help determine the suitability for TMVR and valve sizing.

MAC is often asymmetrically distributed in the posterior aspect of the mitral annulus, but wide variability exists in its extension to the anterior annulus, leaflets, myocardium, and density.^33^ In general, 270 degrees of MAC or surgical bands is sufficient to anchor and seal an implanted valve. Solid MAC provides better anchoring than caseous MAC with core liquefaction necrosis. Objective quantification of MAC using a MAC score (incorporating average calcium thickness, circumferential degrees of calcification involved, calcification at trigones, and calcification at leaflets) has been evaluated, and a MAC score ≤ 6 was found to be independently associated with valve embolization and migration.^34^

The scheme of determining the valve size in ViMAC and ViR depends on the rigidity of the landing zone. In a rigid landing zone, with continuous and solid MAC or a rigid circumferential surgical ring, the valve is sized to fill the widest intercommissural distance.^11^ In a nonrigid landing zone, with noncontinuous and caseous MAC or nonrigid surgical rings and bands, the valve is sized based on annulus area with ≥ 20% oversize.^11^ This allows a compliant nonrigid landing zone to deform and fit circular transcatheter heart valves to minimize the paravalvular leak. In addition, the fabric skirt of a 29-mm SAPIEN 3 can be modified using polytetrafluorethylene to reduce the risk of paravalvular leak.^35^ The valve size in ViV is determined by the inner diameter and rigidity (suitable for valve fracture or not) of the failed surgical bioprosthesis. It is important to note that a small bioprosthesis may produce patient prosthesis mismatch following TMVR, since surgical bioprostheses with an internal diameter < 22 mm were associated with a high risk of mitral stenosis following TMVR in vitro.^36^

### Virtual Implantation of Transcatheter Heart Valve

**Figure 1:**
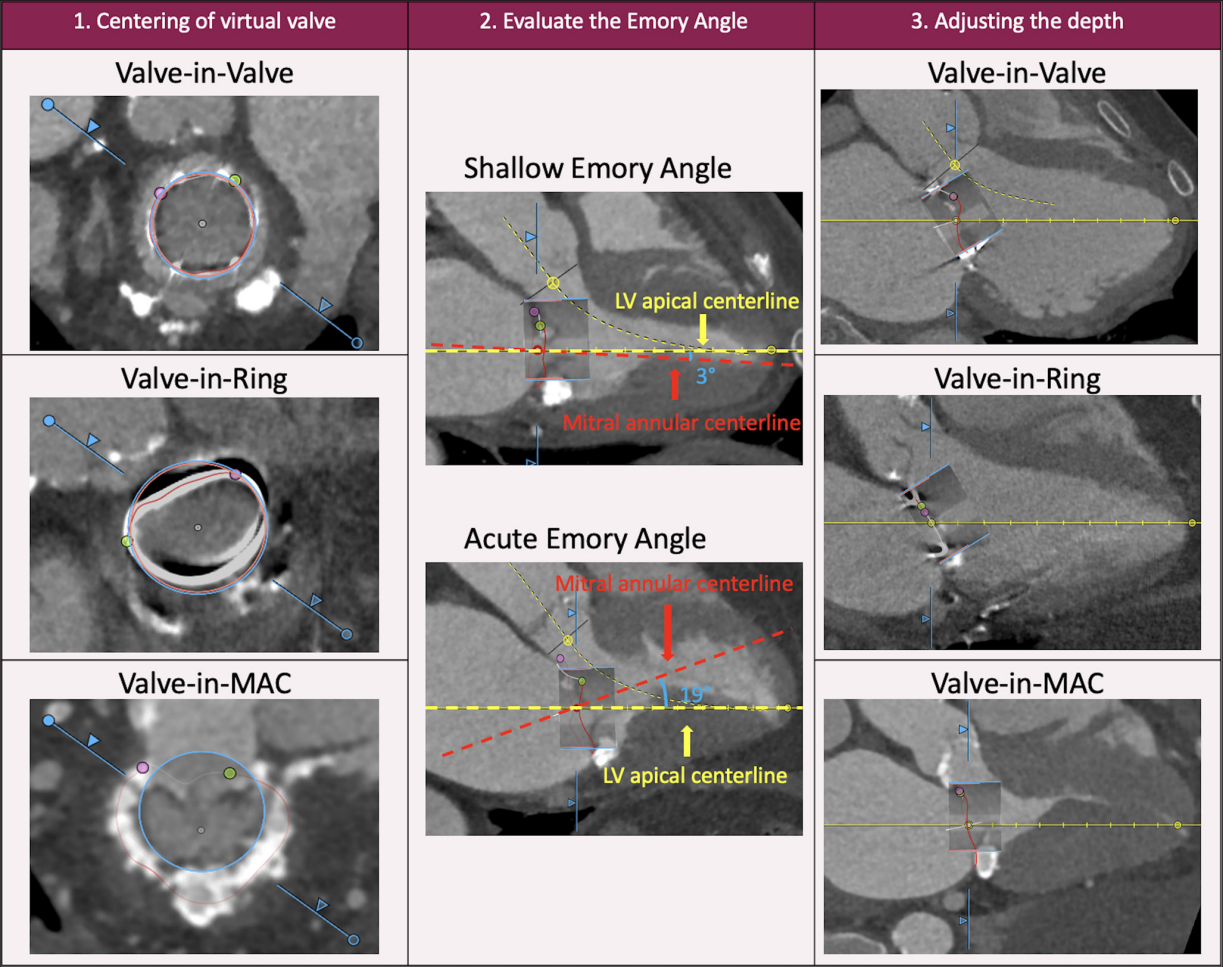
Steps for virtual valve implantation in valve-in-valve, valve-in-ring, and valve-in-MAC transcatheter mitral valve replacement. Step 1 (left column): The mitral annulus is traced along the failed bioprosthetic valve, surgical ring/band, or MAC, and a virtual valve is implanted at the center where the valve is expected to land. Step 2 (middle column): The left ventricular and mitral annular centerline are compared to evaluate the Emory angle. Lastly, the depth is adjusted. Step 3 (right column): In the case of valve-in-valve TMVR, the depth is adjusted so that the transcatheter heart valve aligns with the post. For valve-in-ring and valve-in-MAC, the depth is adjusted to ensure the external skirt contacts the entire landing zone. MAC: mitral annular calcification; TMVR: transcatheter mitral valve replacement

Virtual implantation of the SAPIEN device consists of three steps (Figure 1). First, the virtual valve is centered in the mitral short axis at the narrowest level, where it contacts the calcification (ViMAC), surgical ring (ViR), or bioprosthetic valve (ViV). Next, the virtual valve is tilted to align with the left ventricular apex centerline. This is because the SAPIEN device is delivered along the stiff wire that rests at the left ventricular apex. This process identifies the angle between the mitral annular and left ventricular apex centerline (Emory angle). Emory angles ≥ 15 degrees for SAPIEN 3 and ≥ 20 degrees for SAPIEN 3 Ultra are at risk of significant paravalvular leak and may require additional measures, such as a para-apical rail, to best align the valve.^37^ Finally, the implantation depth is adjusted to ensure that the external skirt contacts the entire landing. This typically results in a 70% to 80% ventricular and a 20% to 30% atrial implantation.

### Assessment of LVOT Obstruction Risk

**Figure 2:**
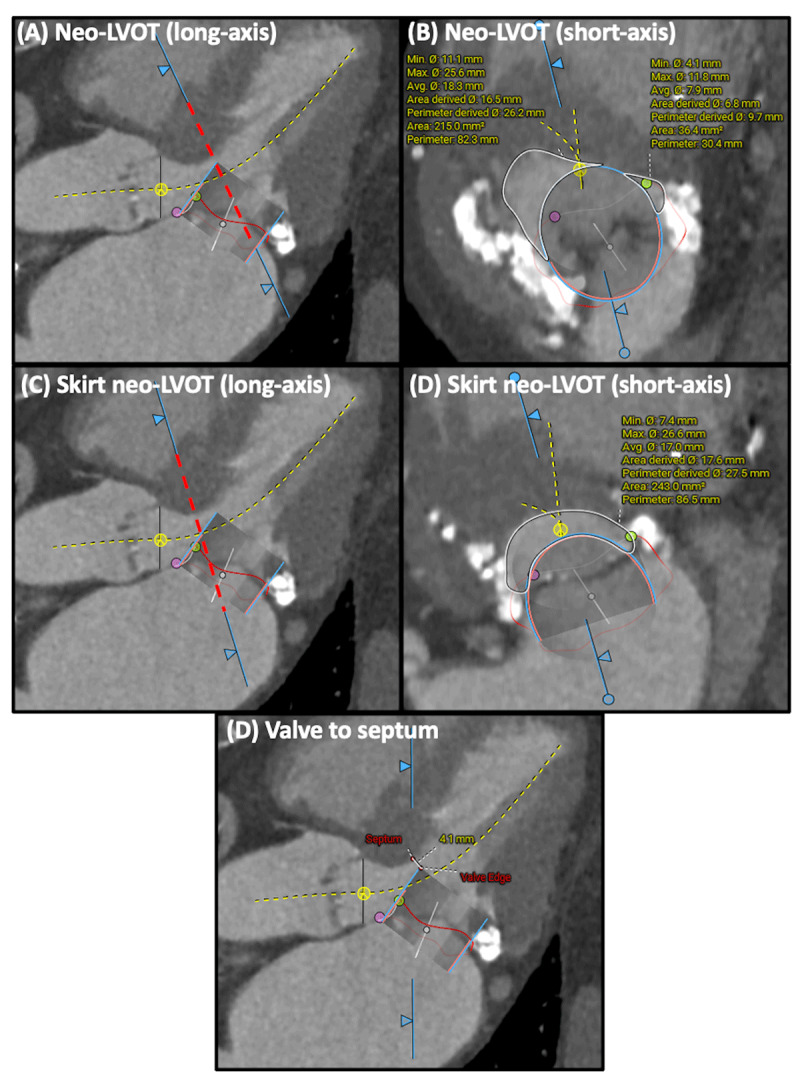
Assessment of left ventricular outflow tract (LVOT) obstruction risk. Risk of LVOT obstruction is assessed by measuring neo-LVOT (**A**: long-axis; **B**: short-axis), skirt neo-LVOT (**C**: long-axis; **D**: short-axis), and valve to septum length (optional) (D).

TMVR involves significant risk of LVOT obstruction, as described above. Risk assessment includes a comprehensive evaluation of the neo-LVOT area, valve-to-septum distance, anterior mitral leaflet length, aorto-mitral angle, septal bulge, and left ventricular size.^38,39,40,41,42^ The neo-LVOT area is measured by the narrowest cross-sectional area between the bulging interventricular septum and the most ventricular portion of the virtual valve (Figure 2). Studies have yielded various cutoff values (170 mm^2^ to 189.4 mm^2^) to predict LVOT obstruction after TMVR.^39,40,42^ With a safety margin, a neo-LVOT area ≥ 200 mm^2^ is considered safe to perform TMVR without additional intervention. When a modification to the anterior leaflet (known as LAMPOON, or laceration of the anterior mitral valve leaflet to prevent LVOT obstruction)^44^ is performed to prevent LVOT obstruction, the neo-LVOT shifts towards the base of the implanted valve at the level of the SAPIEN skirt, creating “skirt” neo-LVOT (Figure 2). A skirt neo-LVOT < 150 mm^2^ to 180 mm^2^ may still produce LVOT obstruction despite a successful LAMPOON.^43,44^ A more simplified measurement of the distance from the distal edge of the virtual valve to the basal septum with a cutoff < 5.5 mm also appears to be a predictor of LVOT obstruction (Figure 2).^39^ Although anterior mitral leaflet length alone has not been reported as an independent risk factor for LVOT obstruction, cases of overhanging leaflets causing central mitral regurgitation through leaflet interference or dynamic LVOT obstruction via the Venturi effect have been observed when leaflet length is longer than the SAPIEN valve height.^41^

### Measures to Prevent LVOT Obstruction

Cases at risk of LVOT obstruction require adjunctive measures before proceeding with TMVR. The approach can be classified into anterior leaflet modification and septal reduction strategies.

**Figure 3:**
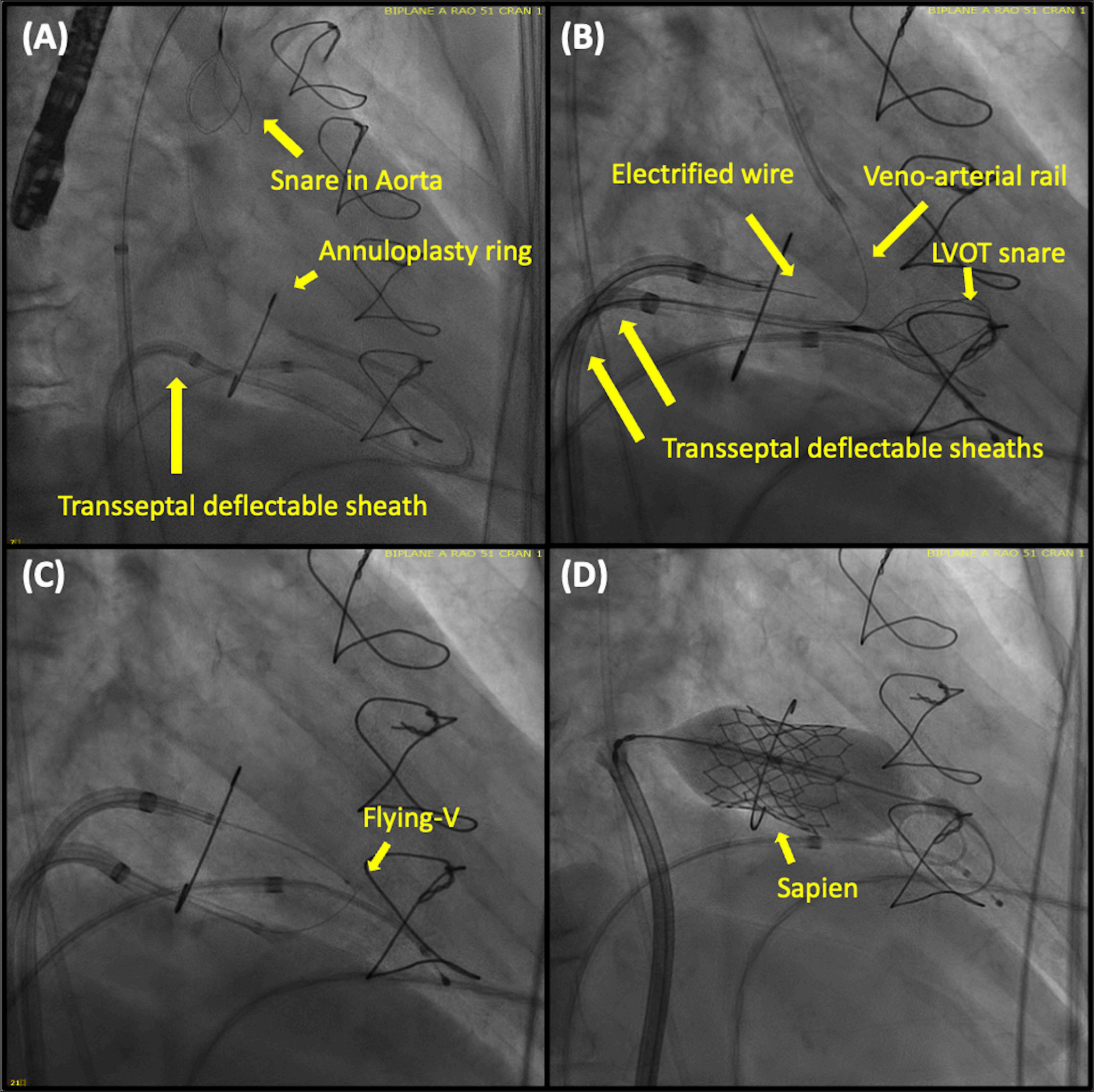
Antegrade laceration of the anterior mitral valve leaflet to prevent left ventricular outflow tract obstruction (LAMPOON). **(A)** A balloon-wedge end-hole catheter is advanced into the left ventricle (LV) via a transseptal deflectable sheath. A guidewire is then advanced through this into the aorta and ensnared by the multiloop snare, creating a venoarterial rail. This step allows for the stabilization of the LV snare. **(B)** The electrified guidewire is delivered through a second transseptal deflectable sheath and is used to traverse the anterior mitral leaflet before being ensnared by a multiloop snare positioned in the LV. **(C)** The flying-V (a kinked and denuded guidewire that allows electrosurgical laceration) is advanced to the anterior mitral leaflet, and a laceration is made. **(D)** The transcatheter heart valve is then deployed.

Laceration of the anterior mitral valve leaflet to prevent LVOT obstruction is an adjunctive transcatheter electrosurgical procedure performed immediately before TMVR.^45^ It involves traversing and splitting the anterior mitral leaflet along the midline. This allows blood to flow through the open cells of the SAPIEN frame (which otherwise would have been covered by the native anterior mitral leaflet) into the LVOT during systole. Several variants have been described, with antegrade LAMPOON currently favored due to shorter procedure time and reproducibility among different operators (Figure 3).^46^ LAMPOON should be considered in patients with neo-LVOT < 200 mm^2^. In the Investigational Device Exemption trial, LAMPOON was successful in all patients, and LVOT gradient < 30 mm Hg following TMVR was achieved in 97%, with LVOT obstruction occurring in only one patient who had a skirt neo-LVOT > 150 mm^2^.^45^

**Figure 4:**
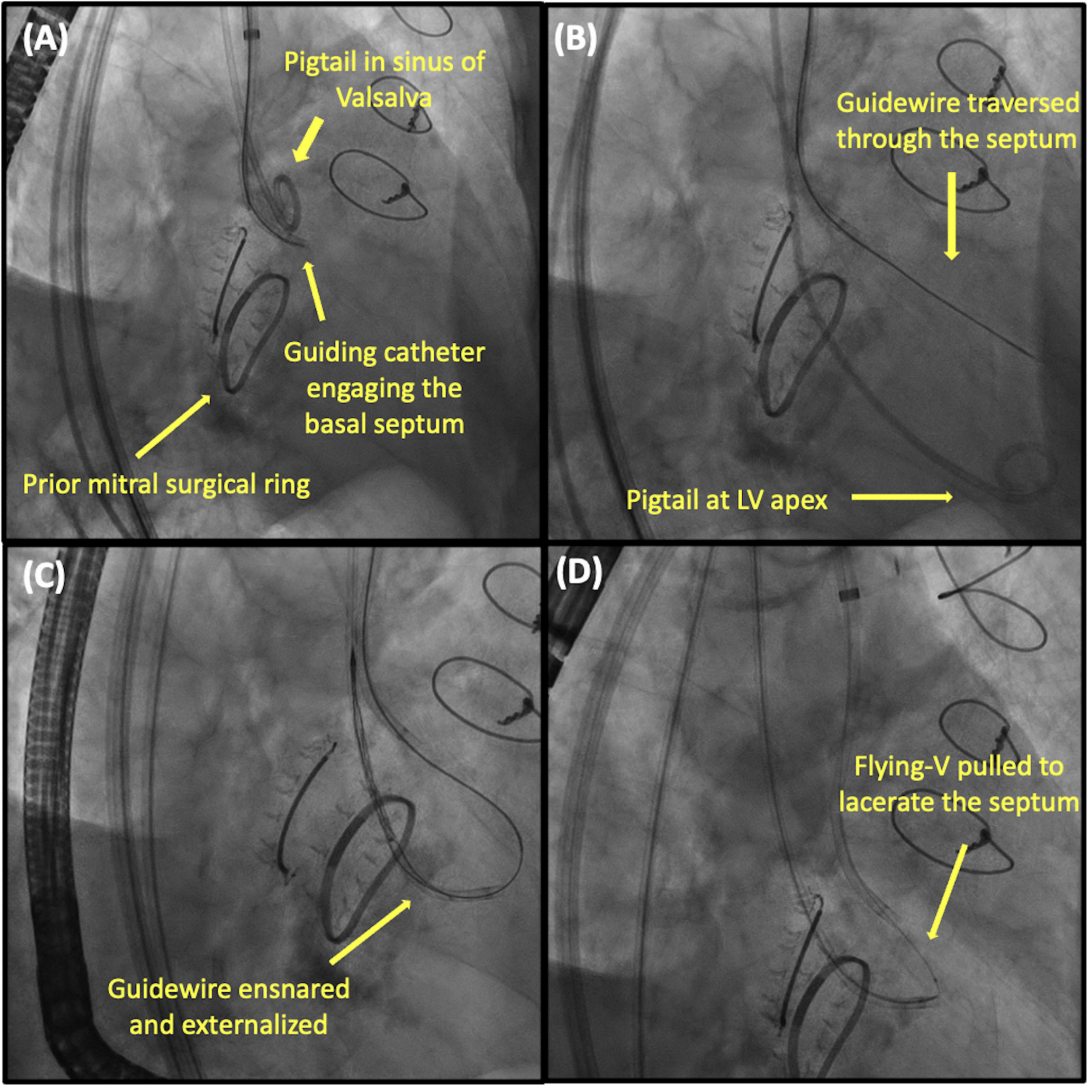
Transcatheter septal myotomy by septal scoring along the midline endocardium. **(A)** A retrograde guide catheter is advanced to engage the basal septum. **(B)** A stiff guidewire is then advanced through this to traverse the septum until it re-enters the left ventricle (LV). **(C)** The guidewire is ensnared by a multiloop snare positioned at the left ventricular apex. **(D)** The flying-V is then advanced and electrified to lacerate the septum.

Septal reduction therapy should be considered in patients with skirt neo-LVOT < 180 mm^2^ since they are at risk of LVOT obstruction despite LAMPOON.^11^ Preemptive alcohol septal ablation has been investigated and shown promising results with an increase in neo-LVOT of 111.2 mm^2^, allowing TMVR in all patients at risk of LVOT obstruction included in the first-in-human study.^47^ The inherent risk of this procedure involves reliance on an adequate septal perforating artery in an anatomically favorable position (at the septal bulge) and a relatively high rate of permanent pacemaker implantation ranging from 16% to 35%.^47,48^ Radiofrequency ablation has been described as an alternative that does not rely on a septal perforating artery. In a case series of four patients, it successfully prevented LVOT obstruction after TMVR in all patients with a tradeoff of 100% permanent pacemaker implantation.^49^ Transcatheter septal myotomy by septal scoring along the midline endocardium has been developed and shown successful results in a first-human report (Figure 4).^50^

## Clinical Outcomes

Details of clinical outcomes of major studies and registries are summarized in Table 1.^17,18,19,20,21,22,51,52,53,54^ Clinical outcomes largely depend on the clinical scenario, with ViMAC having the worst. Large registry-based data reports a 30-day mortality of 4.7% to 8.1% in ViV, 6.7% to 11.5% in ViR, and 11.1% to 34.5% in ViMAC.^10,20,21,25^ More recent data from the MITRAL trial now demonstrates improvement in 30-day mortality (ViV 3.3%, ViR 6.7%, ViMAC 16.7%), 1-year mortality (ViV 3.3%, ViR 23.3%, ViMAC 34.5%), and 2-year mortality (ViV 6.7%; ViR 50%; ViMAC 39.3%).^18,19,22,55^ This improvement is corroborated in the STS/TVT registry^10^ and is thought to be multifactorial, including better patient selection, improved operator experience, the transition from a transapical to transeptal approach, and development of measures to decrease the risk of LVOT obstruction. However, mortality after ViMAC remains poor, reflecting both a challenging patient population and the complexity of the intervention.

**Table 1:** Clinical outcomes of major studies.


	PATIENT NUMBER	TECHNICAL SUCCESS	LVOT OBSTRUCTION	VALVE EMBOLIZATION	NEED FOR SECOND VALVE	CONVERSION TO SURGERY	MORTALITY	STROKE

**Valve-in-Valve**								

**Urena 2018^21^**	34	94.1%	2.9%	2.9%	2.9%	0.0%	30-day: 5.9%; 1-year: 13.2%; 2-year: 29.4%	30-day: 5.9%

**Yoon 2019^20^**	322	94.4%	2.2%	0.9%	2.5%	0.9%	30-day: 6.2%; 1-year: 14.0%	30-day: 2.3%

**STS/ACC TVT registry (2020)^52^**	1,529	96.8%	0.9%	0.3%	0%	0.9%	30-day: 5.4%; 1-year: 16.7%	30-day: 1.1%; 1-year: 3.3%

**VIVID (2021)^53^**	857	93.5%	1.8%	2.4%	2.8%	-	30-day: 6.5%; 1-year: 13.8%; 4-year: 37.5%	Procedural: 1.4%

**MITRAL (2021, 2022)^22,54^**	30	100%	0.0%	0.0%	0.0%	0.0%	30-day: 3.3%; 1-year: 3.3%; 2-year: 6.7%	30-day: 3.3%; 1-year: 6.7%; 2-year: 6.7%

**Valve-in-Ring**								

**Urena 2018^21^**	30	80.0%	0.0%	3.4%	16.7%	6.7%	30-day: 6.7%; 1-year: 12.7%; 2-year: 24.5%	30-day: 0.0%

**Yoon 2019^20^**	141	80.9%	5.0%	1.4%	12.1%	2.8%	30-day: 9.9%; 1-year: 30.6%	30-day: 0.0%

**STS/ACC TVT registry (2020)^55^**	123	82.9%	4.9%	2.4%	7.3%	2.4%	30-day 11.5%	30-day: 0.0%

**VIVID (2021)^53^**	222	82.0%	5.9%	7.0%	10.1%	-	30-day: 8.6%; 1-year: 23.2%; 4-year: 50.3%	Procedural: 0.5%

**MITRAL (2021, 2022)^19,54^**	30	66.7%	0.0%	0.0%	20%	0.0%	30-day: 6.7%; 1-year: 23.3%; 2-year: 50%	30-day: 3.3%; 1-year: 3.3%; 2-year: 3.6%

**Valve-in-MAC**								

**Guerrero 2018^17^**	116	76.7%	11.2%	4.3%	14.7%	3.4%	30-day: 25.0%; 1-year: 53.7%	30-day: 4.3%; 1-year: 6.6%

**Urena 2018^21^**	27	77.7%	7.4%	0.0%	22.2%	0.0%	30-day: 11.1%; 1-year: 41.7%; 2-year: 58.4%	30-day: 7.4%

**Yoon 2019^20^**	58	62.1%	39.7%	6.9%	5.2%	8.6%	30-day: 34.5%; 1-year: 62.8%	30-day: 3.9%

**STS/ACC TVT registry (2020)^55^**	100	74.0%	10.0%	3.0%	14.0%	2.0%	30-day: 21.8%	30-day: 6.3%

**MITRAL (2021, 2022)^18,54^**	31	74.2%	9.7%	0.0%	3.2%	0.0%	30-day: 16.7%; 1-year: 34.5%; 2-year: 39.3%	30-day: 6.7%; 1-year: 6.9%; 2-year: 10.7%


ACC: American College of Cardiology; LVOT: left ventricular outflow tract obstruction; MITRAL: mitral implantation of transcatheter valves; STS: Society of Thoracic Surgeons; TVT: transcatheter valve therapy; VIVID: Valve-in-Valve International Data Registry

Other major clinical complications reported at 30-days in the national database include stroke (1.9%), LVOT obstruction (1.9%), mitral valve re-intervention (1.8%), device embolization (0.8%), myocardial infarction (0.5%), and device thrombosis (0.4%).^10^

## Conclusion

Transcatheter ViV, ViR, and ViMAC TMVR using SAPIEN devices are increasingly being used. Unique challenges remain, including the risk of valve migration and embolization, paravalvular leak, LVOT obstruction, valve thrombosis, and uncertainty in longevity. Preprocedural planning and adjunctive procedures play a key role in successful TMVR. Although clinical outcomes after TMVR have improved over the years, mortality after ViMAC remains poor, and further refinement in patient selection and procedural planning is needed.

## Key Points

- The number of valve-in-valve (ViV), valve-in-ring (ViR), and valve-in-mitral annular calcification (ViMAC) transcatheter mitral valve repair (TMVR) performed are increasing and expected to expand even more.
- Complexity of the mitral apparatus poses unique challenges for TMVR, including the risk of valve migration and embolization, paravalvular leak, left ventricular outflow tract (LVOT) obstruction, valve thrombosis, and uncertainty in longevity.
- Preprocedural computed tomography allows careful evaluation of the landing zone, valve sizing, and risk of LVOT obstruction following TMVR.
- Modification of the anterior mitral leaflet or preemptive septal reduction therapy should be planned as an adjunct to TMVR in cases of high LVOT obstruction risk after TMVR.
- Clinical outcomes after ViV, ViR, and ViMAC are improving over time, but 1-year mortality after ViMAC TMVR remains high.
